# Paraspinal muscle oxygenation and mechanical efficiency are reduced in individuals with chronic low back pain

**DOI:** 10.1038/s41598-024-55672-8

**Published:** 2024-02-28

**Authors:** Agathe Anthierens, André Thevenon, Nicolas Olivier, Patrick Mucci

**Affiliations:** 1 Univ. Lille, Univ. Artois, Univ. Littoral Côte d’Opale, ULR 7369 - URePSSS - Unité de Recherche Pluridisciplinaire Sport Santé Société, 59000 Lille, France; 2 Service de Médecine Physique et de Réadaptation Fonctionnelle, CHRU Lille, Lille, France

**Keywords:** Exercise, Oxygen consumption, Paraspinal muscles, Low back pain, Blood volume, Physiology, Musculoskeletal system, Pain

## Abstract

This study aimed to compare the systemic and local metabolic responses during a 5-min trunk extension exercise in individuals with chronic low back pain (CLBP) and in healthy individuals. Thirteen active participants with CLBP paired with 13 healthy participants performed a standardised 5-min trunk extension exercise on an isokinetic dynamometer set in continuous passive motion mode. During exercise, we used near-infrared spectroscopy to measure tissue oxygenation (TOI) and total haemoglobin-myoglobin (THb). We used a gas exchange analyser to measure breath-by-breath oxygen consumption (V̇O_2_) and carbon dioxide produced (V̇CO_2_). We also calculated mechanical efficiency. We assessed the intensity of low back pain sensation before and after exercise by using a visual analogue scale. In participants with CLBP, low back pain increased following exercise (+ 1.5 units; *p* < 0.001) and THb decreased during exercise (− 4.0 units; *p* = 0.043). Paraspinal muscle oxygenation (65.0 and 71.0%, respectively; *p* = 0.009) and mechanical efficiency (4.7 and 5.3%, respectively; *p* = 0.034) were both lower in participants with CLBP compared with healthy participants. The increase in pain sensation was related to the decrease in tissue oxygenation (R^2^ = − 0.420; *p* = 0.036). Decreases in total haemoglobin-myoglobin and mechanical efficiency could involve fatigability in exercise-soliciting paraspinal muscles and, therefore, exacerbate inabilities in daily life. Given the positive correlation between tissue oxygenation and exercise-induced pain exacerbation, muscle oxygenation may be related to persisting and crippling low back pain.

## Introduction

Chronic low back pain (CLBP) is a centuries-old health preoccupation due to its high prevalence and physical, psychosocial and economic repercussion. In 2018, the World Health Organisation called for action to address the challenges associated with preventing disabling low back pain^1^. CLBP does not only lead to pain^2^. It is also associated with physical^3^ and psychosocial limitations^4^. These limitations lead to a vicious circle, in which physical deconditioning implies a reduction in physical and social activities, and an exacerbation of pain^5^.

One of the major physical impairments perpetuated by the deconditioning circle is the excessive fatigability of the paraspinal muscles (iliocostalis, longissimus, spinalis and multifidus)^6^. In fact, this phenomenon has been widely reported in the literature through weak performance during prolonged exercise and myoelectric manifestations of fatigue assessed by electromyography^7^. Paraspinal muscle fatigability implies functional disability in daily life and a deterioration in quality of life, as these muscles are constantly involved in maintaining posture^8^.

The excessive paraspinal muscle fatigability and low back pain that characterise CLBP may be related to an alteration in the aerobic contribution to exercise. In fact, limiting the aerobic contribution would lead to an increase in the anaerobic contribution. The anaerobic contribution is associated with the accumulation of metabolites, including ATP, lactate and H^+^. Previous studies have shown that this metabolite accumulation can lead to muscle fatigue, pain sensation^9^ and induce muscle hyperalgesia^10^.

Several elements suggest that the aerobic contribution to exercise may be impaired in individuals with CLBP: On the one hand, previous studies revealed that compared with healthy individuals, individuals with CLBP have fewer oxidative fibres in the paraspinal muscles, and these fibres have a reduced diameter^11^. On the other hand, paraspinal muscle contraction may impede blood flow due to an increase in intramuscular pressure, compromising muscle oxygenation^12^. This phenomenon could be exacerbated in individuals with CLBP^13^. Such alterations may limit the aerobic contribution to exercise and thus contribute to worsening the limiting symptoms of CLBP.

Researchers have investigated this hypothesis by measuring muscle oxygenation with near-infrared spectroscopy (NIRS). However, the results are conflicting: some authors have found a difference in low back muscle oxygenation in individuals with CLBP compared with healthy individuals^14,15^, while others have found no difference in low back muscle oxygenation or local blood volume^16,17^. These inconsistencies must be the result of differences in protocol design. The tests can consist of static or dynamic contractions (e.g. the Sorensen test^14^ versus a lifting task^15^), with the intensity based on anthropometric data or on the individual’s strength (e.g. based on height and weight^16^ versus the number of repetitions^15^). In addition, inconsistencies may be due to the physical activity level of the participants, which has not been reported consistently, although it deserves consideration. It influences the response to exercise, regardless of the level of low back pain^14,18^. Thus, it is unclear whether the changes in muscle oxygenation in individuals with CLBP are solely due to physical deconditioning, or whether the alterations persist in active individuals with CLBP.

Previous studies have another limitation: the aerobic contribution to exercise is only assessed using NIRS techniques, analysing muscle responses, without assessing systemic responses. However, systemic responses—such as cardiorespiratory responses—may also reflect the aerobic contribution to exercise. There is a need to investigate metabolic responses at different levels (systemic and muscle) to clarify the physiopathology of CLBP. The use of a gas exchange analyser is a relevant way to assess systemic responses to exercise, and to calculate energy cost^19^ and onset kinetics^20^. Alteration of any of these variables could be attributed to a limitation in the aerobic contribution to exercise^21^, resulting in muscle fatigability. To our knowledge, gas exchange has only been analysed in individuals with CLBP during maximal incremental whole-body exercise (i.e. cycling). However, the relevance of this type of exercise can be questioned. Whole-body exercise involves many muscles other than those of the lower back^22^, and the maximum exercise intensity is rarely reached in everyday life^23^. A trunk extension exercise is more relevant to study paraspinal muscle metabolic responses to exercise because these muscles are involved in trunk extension^24^. Moreover, prolonged submaximal trunk extension exercise is more useful to specifically solicit the lower back and to replicate a task of daily life such as lifting, carrying or bending^25,26^. Such an exercise can be set using an isokinetic dynamometer. Isokinetic dynamometry offers the possibility to standardise exercise in terms of intensity, range of motion and individual position. The movement occurs in one plan, partially imposing the movement pattern.

To date, there has not been a comparison of systemic and local metabolic responses of the lower back in healthy individuals and individuals with CLBP during standardised submaximal exercise. The purpose of this study was to analyse systemic and local metabolic responses during prolonged trunk extension exercise in individuals with CLBP. Our hypothesis was that participants with CLBP would present reduced paraspinal muscle endurance and, as a result of altered metabolic responses, would have slower V̇O_2_ kinetics, lower mechanical efficiency and lower muscle oxygenation compared with matched healthy participants.

## Methods

### Ethical approval

The study was approved by the Committee for the Protection of Persons (CPP Nord Ouest IV—ID OXYLOM 2015_58) and by the French National Agency for Medicine and Health Product Safety (ANSM—IDRCB: 2016-A01151-50). The procedures were performed in accordance with the Declaration of Helsinki and its later amendments. Each participant signed a written informed consent form.

### Population

Individuals with nonspecific CLBP were included in this study. They were invited by a physician to voluntarily participate in this study. To be included, they had to have had low back pain for at least 3 months. People with a body mass index over 25 kg m^−2^ or under 18.5 kg m^−2^ were not included in the study. Participants had to be physically active, in accordance with the World Health Organization^27^ (i.e. at least 150 min of moderate-intensity aerobic physical activity throughout the week or at least 75 min of vigorous-intensity aerobic physical activity throughout the week or an equivalent combination of moderate- and vigorous-intensity activity). The level of physical activity was assessed in an interview, and then quantified using the Baecke questionnaire^28^. It assesses physical activity during work/occupational activities, during leisure time, during active commuting, and during sports activities. Each participant with CLBP was matched with a healthy volunteer in terms of age, weight, height, physical activity level and smoking status (based on the number of cigarettes consumed per). Any individual with a history of cardiovascular, metabolic, respiratory or neurological disease was excluded from the study.

The sample size was estimated with a paired t-test in the Sigmastat 3.5 software. We considered previously published results evaluating muscle oxygenation in individuals with CLBP and a control group. The values were 12.3 ± 3.0 and 9.3 ± 2.9, respectively^29^. The alpha level used was 0.05 and the fixed power level was 90%. Thus, the sample size for each group was estimated to be 12 participants. As the study protocol was spread over two visits, we anticipated that 10% of the participants would leave the study before the end of the protocol or withdraw their consent, so we aimed to include 14 participants in each group.

### Procedures

The protocol used for the experiment consisted of two visits. During the first visit, the inclusion and exclusion criteria were checked by a physician and anthropometric measurements collected. Each participant performed the Sorensen test^30^ to assess paraspinal muscle endurance. In this test, the participant is positioned prone on a table. The lower part of the body (below the iliac crest) is immobilised. The upper part of the body is out of the table. The test consists of keeping the upper body horizontal, aligned with the lower body. The test was stopped when the participant reached 150 s (corresponding to a higher value than the normative data)^31^.

The second visit was in the same week, at least 24 h after the first visit. During this visit, the participants performed trunk flexion and extension exercises on an isokinetic dynamometer (Con-trex TP-1000, CMV AG, Suisse). Each participant was placed on the dynamometer as described in the manufacturer’s instructions: briefly, the participant was upright, with the knees slightly flexed and a popliteal pad directly behind the patella. The body was attached via a thigh pad, a tibial pad, a scapular pad and a pelvic belt. Once attached and upright, the trunk was tilted slightly forward or backward to define the anatomical zero position. This was defined as the position in which the participant felt neutral. The participant stood in this position during rest periods.

During the exercises, the axis of rotation was placed 3.5 cm below the top of the iliac crest. The range of motion was set at 70°, from − 5° extension to + 65° flexion. During each exercise, the participant was asked to perform trunk extensions, while flexions were passive (using the continuous passive motion mode). The velocity was set at 30° s^−1^ for passive flexion and 60° s^−1^ for trunk extension. After performing three maximal trunk extensions to assess peak torque (a relevant indicator of muscle strength)^32^, the participants were asked to perform a 5-min submaximal exercise session. The intensity was set at 80 Nm. Submaximal intensity dosage was via real-time visual feedback, which continuously showed the development of torque on a screen. The exercise duration and intensity were set at an absolute intensity achievable by all individuals in order to fully engage aerobic metabolism, reflected by a steady-state V̇O_2_ during exercise. The absolute intensity was chosen to replicate an everyday lifting task.

During this exercise, the total work developed during trunk extension was calculated by the dynamometer. Before and after the exercise, a visual analogue pain scale was used to evaluate the intensity of pain sensation in the lower back (rated from 0 corresponding to ‘No pain’ to 10 corresponding to ‘Worst pain imaginable’). In addition, muscle oxygenation and the cardiorespiratory response were recorded and analysed using NIRS and a gas exchange analyser, respectively.

### NIRS measurements

NIRS was used to assess paraspinal muscle oxygenation continuously as described previously^33^. This technique is based on the light absorption properties of oxy-haemoglobin/myoglobin (HbO_2_), which absorbs light at 850 nm, and of deoxy-haemoglobin/myoglobin (HHb), which absorbs light at 760 nm. The NIRS device (Portamon Artinis, Zetten, the Netherlands) covered with plastic film to avoid sweat accumulation was used during exercise. The device was positioned vertically, parallel to the spine. The middle of the device was positioned at the level of the third lumbar vertebra. The device was positioned so that the light emitters and receiver were 3 cm from the spinous process of the vertebra. The distance between the light source and the receiver used was 40 mm. In accordance with the manufacturer’s instructions and previous work^16,34^, the differential pathlength factor applied was 4. This factor takes into account the scattering of light within the tissues.

The values of HbO_2_ and HHb were recorded for 2 min at rest, and then during the 5-min exercise. After recording, the HbO_2_ and HHb values were used to calculate the total haemoglobin/myoglobin (THb = HbO_2_ + HHb), which is a good reflection of the microvascular volume change. The values of HbO_2_, HHb and THb were kept for analysis. The values collected at rest were averaged to obtain HbO_2(0)_, HHb_(0)_ and THb_(0)_. All the data obtained during the exercise were normalised by the resting values to obtain ΔHbO_2_, ΔHHb and ΔTHb.

$$\Delta {\text{HbO}}_{{2}} = {\text{HbO}}_{{2}} - {\text{HbO}}_{{{2}(0)}}$$

$$\Delta {\text{HHb}} = {\text{HHb}} - {\text{HHb}}_{(0)}$$

$$\Delta {\text{THb}} = {\text{THb}} - {\text{THb}}_{(0)}$$

Normalisation of these data is necessary because they are influenced by individual factors (i.e. adipose tissue, skin perfusion, melanin contribution and the heterogeneity of blood flow in muscle)^35^. The tissue oxygenation index (TOI) was also analysed. The NIRS device calculates this index by utilising the spatially resolved spectroscopy technique. The TOI does not require normalisation with the rest value because it is expressed as a percentage, making it a good tool for inter-subject comparison^36,37^. ΔTOI (ΔTOI = TOI_5-min_ − TOI_(0)_) was also calculated to determine a potential relationship between the change in the TOI and the change in the pain intensity (ΔPain = Pain_post-ex_ − Pain_pre-ex_) following exercise.

### Pulmonary gas exchange measurements

A gas exchange analyser (Cortex Metamax 3B, Leipzig, Germany) coupled with a heart rate (HR) monitor (Polar Electro T31, Finland) were used to measure V̇O_2_, V̇CO_2_, respiratory frequency (Fr), tidal volume (Vt) and HR. Before each measurement, the device was calibrated following the manufacturer’s guidelines. After acquisition of the measurements, the minute ventilation (V̇E = Fr × Vt) and respiratory exchange ratio (RER = V̇CO_2_/V̇O_2_) were calculated, as well as the energy expenditure, according to the formula of Brouwer^38^:

$${\text{Energy}}\;{\text{expenditure}}\;\left( {\text{J}} \right) = [({3}.{869} \times {\dot{\mathrm{V}}}{\text{O}}_{{2}} ) + ({1}.{195} \times {\text{V}}{{\text{CO}}}_{{2}} )] \times \left( {{4}.{186}/{6}0} \right) \times {1}000$$

The mechanical efficiency was calculated as described by Moseley and Jeukendrup^38^:

$${\text{Mechanical}}\;{\text{efficiency}}\;\left( \% \right) = \left[ {{\text{Work rate }}\left( {{\text{J s}}^{{ - {1}}} } \right)/{\text{Energy expended }}\left( {{\text{J s}}^{{ - {1}}} } \right)} \right] \times {1}00$$

The V̇_O_2 values collected breath by breath were interpolated to produce one value per second. The onset kinetics was then calculated using a mono-exponential model, V̇O_2(t)_ = V̇O_2(0)_ + A(1 − e ^−(t−TD)/τ^), in which V̇O_2(t)_ represents the oxygen consumption at any time during exercise, V̇O_2(0)_ represents the oxygen consumption at rest, A represents the amplitude between V̇O_2(0)_ and V̇O_2_ at steady state, t represent the time of exercise, TD represents the time delay, and τ represents the time constant (i.e*.* the time required to reach 63% of the steady state). The mean response time (corresponding to the sum of the delay and the time constant) and amplitude were collected.

### Statistical analysis

The Sigmastat 3.5 software was used for statistical analysis. The data are expressed as the mean (standard deviation [SD]), except for ΔHbO_2_, ΔHHb, ΔTHb and TOI, which are expressed as the mean (standard error [SE]) because of the large SDs. A *p* value < 0.05 was considered to indicate a statistically significant difference.

The holding time during the Sorensen test, the peak torque, the mechanical efficiency and the V̇O_2_ onset kinetics (i.e. amplitude and mean response time) were compared between groups using a paired t-test or a Wilcoxon signed-rank test (if the data did not follow a normal distribution). When the data had a normal distribution, the 95% confidence interval (CI) for the difference in means was calculated. The group effect sizes for the t-tests are described with Cohen’s d, calculated as the difference in the means divided by the pooled SD. The effect size is considered small when d = 0.2, medium when d = 0.5 and large when d = 0.8.

Changes in pain sensation intensity, ΔHbO_2_, ΔHHb, ΔTHb, TOI and cardiorespiratory measures (i.e. V̇O_2_, V̇CO_2_, HR, V̇E and RER) during exercise were compared using a two-way analysis of variance (group effect × time effect). When there were differences, a post hoc Bonferroni test was applied. Group effect sizes for analyses of variance are described using the eta squared (η^2^)^39^, calculated as the sum of squares of an effect divided by the total sum of squares. The effect size is small when η^2^ = 0.01, medium when η^2^ = 0.06 and large when η^2^ = 0.14.

The relationship between ΔPain and ΔTOI was analysed with Pearson correlation coefficients.

## Results

### Participants

Fourteen individuals with CLBP were included and paired with healthy individuals, but one of them did not participate in all the testing sessions, resulting in the exclusion of both the individuals with CLBP and the paired healthy individual from the study. All participants were physically active in their leisure time (walking, weightlifting, swimming, cycling, motorcycling, fitness) or at work (forklift driver, roofer). Two Baecke questionnaires were not completed correctly; therefore, the questionnaire data reported in Table 1 includes 11 participants with CLBP and 11 healthy participants. One participant with CLBP smoked; therefore, he was paired with a healthy participant who smoked the same number of cigarettes per day (12 cigarettes per day). The anthropometric data are reported in Table 1.

**Table 1:** Anthropometric characteristics.

	Individuals with chronic low back pain (n = 13)	Healthy individuals (n = 13)
Sex (M/F)	8/5	8/5
Age (years)	44.1 ± 6.9	42.9 ± 6.8
Height (cm)	175 ± 9	17 ± 7
Body mass (kg)	70.2 ± 11.2	69.1 ± 11.4
Body mass index (kg m^−2^)	22.9 ± 2.0	23.2 ± 2.4
Baecke score	8.2 ± 1.1	8.9 ± 1.4

The data are presented as the mean ± standard deviation. There were no significant differences between the groups.

### Low back muscle endurance and strength

**Figure 1:**
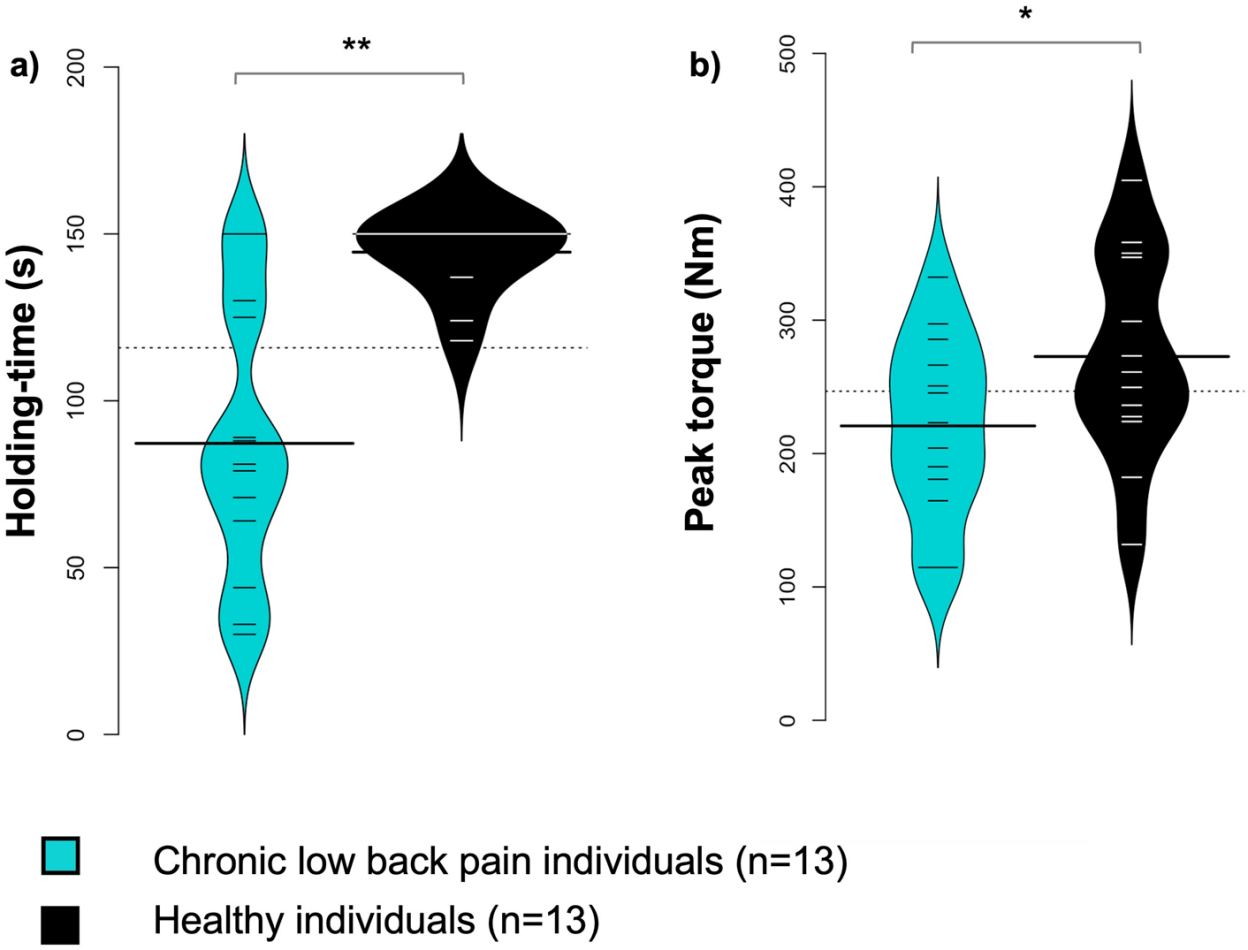
Violin plots showing the holding time during the Sorensen test and peak torque. Bold lines represent the mean of each group; thin lines represent individual values. Significant group effect: * *p* < 0.05 ** *p* < 0.001.

The holding time during the Sorensen test and peak torque developed during the maximal extension exercise were lower in participants with CLBP compared with healthy participants (Sorensen test: 87.23 ± 40.96 and 144.54 ± 11.11 Nm, respectively, *p* < 0.001, 95% CI (− 80.68; 33.94), d = 1.48; peak torque: 220.71 ± 67.43 and 272.72 ± 77.17 s, respectively, *p* = 0.027, 95% CI (− 97.04; − 6.98), d = 0.70) (Fig. 1).

### Five-minute exercise responses

**Figure 2:**
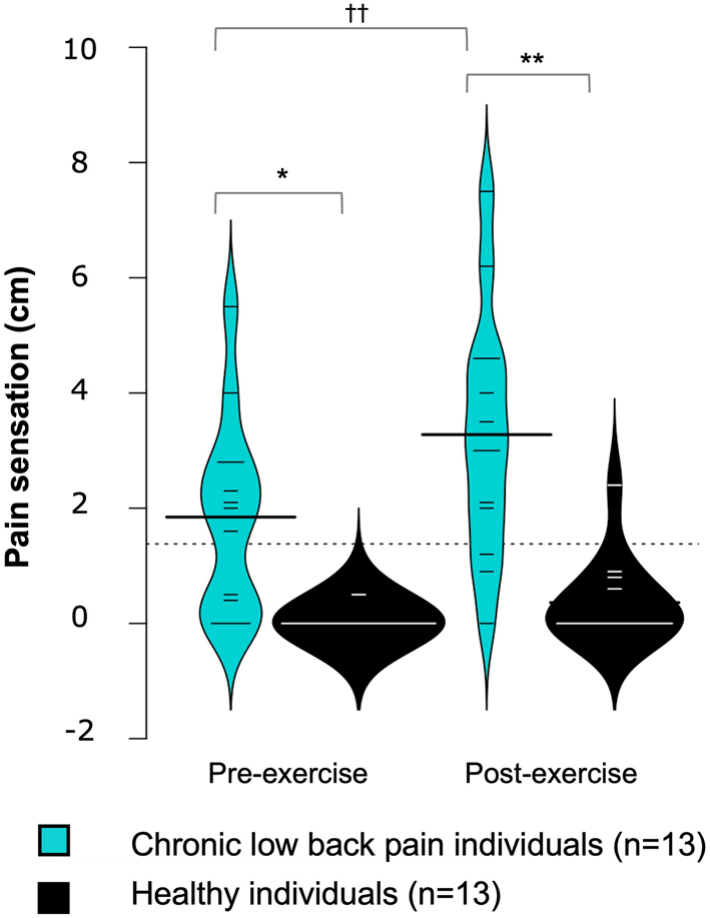
Violin plots showing the intensity of pain sensation in the lower back before and after exercise. Bold lines represent the mean of each group; thin lines represent individual values. Significant group effect: * *p* < 0.05 ** *p* < 0.001. Significant time effect: †† *p* < 0.001.

Regarding pain sensation, there was significant time (*p* < 0.001; η^2^ = 0.06), and group (*p* < 0.001; η^2^ = 0.40) effects. The group × time interaction was significant (*p* = 0.010; η^2^ = 0.02). Pain sensation was greater before and after exercise in participants with CLBP (p = 0.003 and *p* < 0.001, respectively) and it increased following exercise only in the participants with CLBP (*p* < 0.001) (Fig. 2).

Regarding ΔHbO_2_, there was a time effect (*p* < 0.001; η^2^ = 0.070) but not a group effect (*p* = 0.759; η^2^ = 0.003). The group × time interaction was not significant (*p* = 0.967; η^2^ = 0.002). Regarding ΔHHb, there was neither a time (*p* = 0.338; η^2^ = 0.010) nor group (*p* = 0.926; η^2^ = 0.0003) effect. The group × time interaction was not significant (*p* = 0.774; η^2^ = 0.005). Regarding ΔTHb, there was a time effect (*p* = 0.002; η^2^ = 0.040) but not a group effect (*p* = 0.853; η2 = 0.001). The group × time interaction was not significant (*p* = 0.861; η^2^ = 0.004).

**Figure 3:**
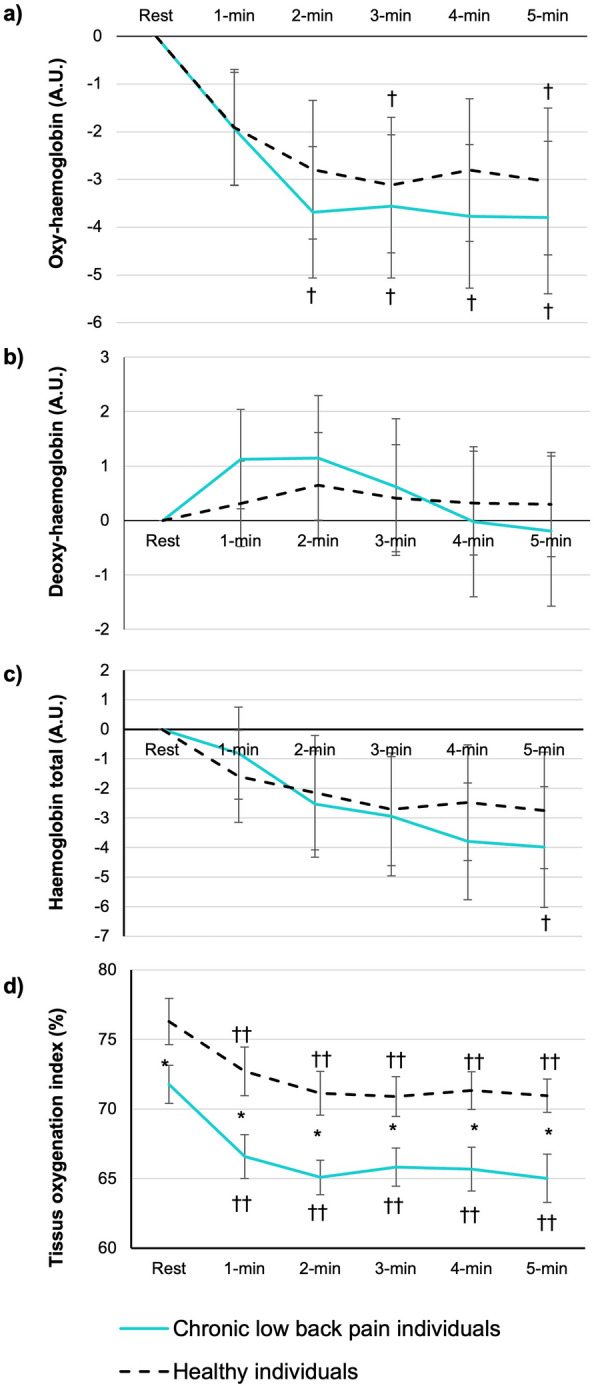
Changes in oxy-haemoglobin, deoxy-haemoglobin, total haemoglobin and the tissue oxygenation index in low back muscles during exercise. The values are presented as the mean ± standard error. Significant group effect: * *p* < 0.05 ** *p* < 0.001. Different from rest: † *p* < 0.05 †† *p* < 0.001.

For TOI, there were significant time (*p* < 0.001; η^2^ = 0.12) and group (*p* = 0.009; η^2^ = 0.20) effects, but the group × time interaction was not significant (*p* = 0.764; η^2^ = 0.002). The pairwise comparisons showed that in both groups, the TOI was lower from the first minute until the last minute of exercise compared with resting values (*p* < 0.001). The TOI was lower in participants with CLBP compared with the healthy participants at rest (*p* = 0.042) and during exercise (first minute: *p* = 0.007; second minute: *p* = 0.008; third minute: *p* = 0.023; fourth minute: *p* = 0.012; fifth minute: *p* = 0.09) (Fig. 3).

For all the cardiorespiratory variables (V̇O_2_, V̇CO_2_, HR, V̇E and RER), there was a time effect (*p* < 0.001) but not a group effect. The mechanical efficiency was lower in the participants with CLBP (*p* = 0.034; d = 0.61). There was no difference between the groups for V̇O_2_ onset kinetics variables (Table 2).

**Table 2:** Cardiorespiratory variables and muscle efficiency at the end of exercise and V̇O_2_ onset kinetics.

	Individuals with chronic low back pain	Healthy individuals	Time effect	Group effect	Effect size (group effect)	95% CI of the difference of the means
V̇O_2_ (L min^−1^)	1.34 ± 0.21	1.25 ± 0.16	< 0.001	0.409	0.00	− 0.1; 0.26
V̇CO_2_ (L min^−1^)	1.32 ± 0.18	1.33 ± 0.18	< 0.001	0.960	0.00	− 0.19; 0.17
HR (beats min^−1^)	135.28 ± 20.80	138.77 ± 23.95	< 0.001	0.669	0.00	− 24.95; 11.40
V̇E (L min^−1^)	46.51 ± 11.52	43.10 ± 6.81	< 0.001	0.196	0.01	− 6.47; 12.91
RER	1.01 ± 0.14	1.06 ± 0.08	< 0.001	0.501	0.01	− 0.14; 0.02
Energy expenditure (J s^−1^)	429.77 ± 91.18	412.90 ± 85.02	–	0.493	0.17	–
Mechanical efficiency (%)	4.7 ± 0.81	5.3 ± 1.16	–	0.034	0.61	–
Amplitude V̇O_2_ (L min^−1^)	1.01 ± 0.23	0.95 ± 0.15	–	0.717	0.11	− 0.18;0.25
Mean response time (s)	59.66 ± 18.38	53.28 ± 24.72	–	0.438	0.24	− 12.89; 27.54

The data are presented as the mean ± standard deviation. The time effect is only reported for variables that were analysed minute by minute. The 95% confidence interval (CI) is only reported for variables that followed a normal distribution.

**Figure 4:**
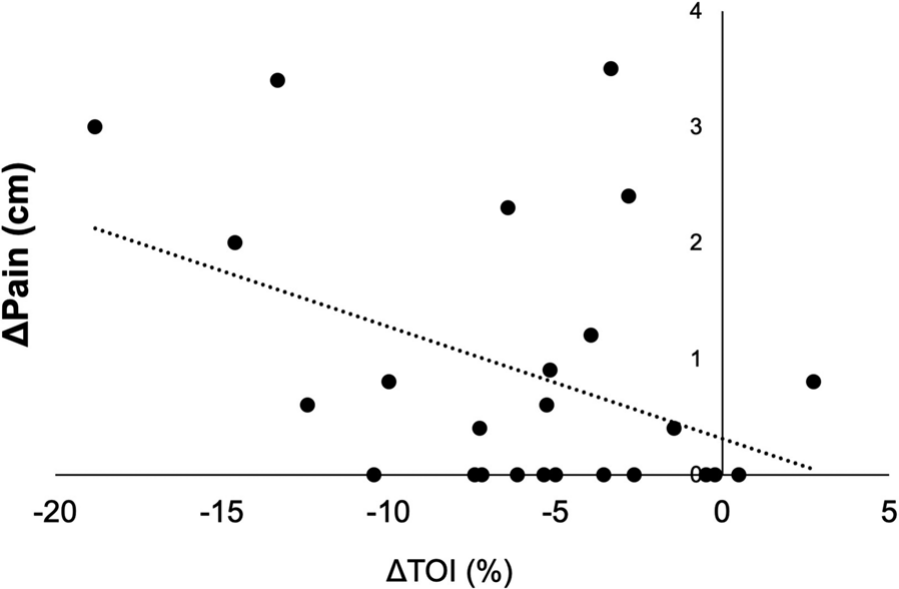
Relationship between changes in pain intensity and changes in the tissue oxygenation index (TOI) induced by exercise (R^2^ = − 0.420; *p* = 0.036).

The correlation between ΔTOI and ΔPain was significantly negative (R^2^ = − 0.420; *p* = 0.036) (Fig. 4).

## Discussion

The aim of this study was to investigate aerobic metabolic adaptations in individuals with CLBP during a trunk extension exercise by measuring systemic and paraspinal muscle responses. For the first time, we have shown reduced mechanical efficiency, in addition to reduced muscle endurance and strength in individuals with CLBP compared with matched healthy individuals. In addition, the TOI was lower in participants with CLBP at rest and during exercise. Moreover, microvascular local blood volume (i.e. THb) decreased during the 5-min trunk extension exercise only in participants with CLBP. These results were independent of physical inactivity or sedentary lifestyle, as the participants were matched on the basis of their physical activity level.

Some outcomes were not different between participants with CLBP and healthy participants, including cardiorespiratory parameters, corroborating the preliminary results discussed in the study by Vrana and colleagues^40^. They suggested that variability in paraspinal muscle metabolic responses to exercise may exist without variability in systemic responses. There was also no difference in V̇O_2_ kinetics. Because V̇O_2_ onset kinetics is mainly dependent on the oxidative capacity of the muscle^41^, our results suggest that it is not altered in the paraspinal muscles of individuals with CLBP. This is reinforced by the lack of difference in ΔHHb during exercise between groups, as it is an indicator of O_2_ extraction^37^.

### Alteration in mechanical efficiency and tissue oxygenation

As previously demonstrated, people with CLBP have reduced holding times during the Sorensen test^14^ and reduced peak torques during trunk extension^42^, demonstrating poor low back muscle endurance and strength. In addition, we have shown for the first time that paraspinal muscle oxygenation is reduced in individuals with CLBP, even at rest. The lower TOI in the standing position may be associated with higher muscle O_2_ consumption. This alteration could be related to functional limitations in usual activities, even in very-low intensity tasks.

During exercise, TOI was even lower, and we found reduced mechanical efficiency. This shows an increase in oxygen cost to perform tasks. An increased metabolic cost may be associated with an impaired physical ability to perform tasks in individuals with CLBP. The combination of these outcomes may explain, at least in part, the reduced functional capacity to perform daily tasks. Moreover, the reduction in the TOI even at rest may be related to functional limitations in usual activities, even during very-low intensity tasks.

Several hypotheses may explain the decrease in mechanical efficiency and the TOI. First, these may be affected by muscle activation. Individuals with CLBP are known to have particular motor patterns, such as altered flexion-relaxation responses to trunk flexion and/or increased activation of the low back muscles while standing^43,44^. In addition, the level of activation of co-agonist and antagonist muscles may differ^17^. These phenomena may be secondary to pain or kinesiophobia, and may alter muscle solicitations, and thus metabolic needs. The increased contribution of the low back muscles in the standing position could explain both the decrease in oxygenation at rest—denoted by the lower TOI—and the decrease in mechanical efficiency.

Second, mechanical efficiency may be affected by haemodynamic responses to exercise. On the one hand, previous work has shown that blood flow restriction can increase energy expenditure^45^, thereby reducing mechanical efficiency. On the other hand, previous studies have shown that contraction of low back muscles increases lower back intramuscular pressure, which can restrict blood flow due to blood vessel crushing^12,46^. In our study, microvascular blood volume restriction in participants with CLBP is supported by a reduction in ΔTHb during exercise. Impaired mechanical efficiency in participants with CLBP could be secondary to increased lower back intramuscular pressure. This view is supported by previous studies reporting lumbar compartment syndrome in individuals with CLBP during exercise^13^. This would contribute to reduced perfusion in the lower back and induce altered muscle oxygenation, as evidenced by the reduction in the TOI in the participants with CLBP, which is influenced by local blood flow^47^. Although exercise-induced intramuscular pressure may explain exercise-induced low back pain, such a chronic phenomenon that persists even at rest has rarely been described, and it appears to be a very rare factor associated with persistent low back pain^48^.

### Relationship between paraspinal pain and oxygenation during exercise

Our results suggest that impaired muscle oxygenation may be associated with low back pain. In response to exercise, ΔTOI may increase due to increased O_2_ consumption, but it may also increase secondarily to blood flow restriction^47^, as suggested by the decrease in ΔTHb in this study. Previous work has indicated the association between blood flow restriction and substance P accumulation in an animal model^46^, and hypoperfusion and hypoxia have already been related to pain sensation in humans^49,50^. The potential relationship between pain and altered haemodynamics and/or oxygenation is supported by the negative correlation between ΔTOI and ΔPain, and it deserves further investigation.

### Practical implications

People with CLBP showed reduced endurance-time, mechanical efficiency and muscle oxygenation, without cardiorespiratory alteration to exercise. The muscle oxygenation index was negatively correlated with the pain sensation intensity in the low back muscles. All of these adaptations suggest that physical disability could be associated with altered muscle metabolic adaptations to exercise. It may be necessary to stimulate muscle aerobic metabolism to reduce muscle fatigue and muscle pain. High-volume resistance training could be relevant to improve local aerobic adaptations. This approach could be more effective than traditional resistance exercise, traditional aerobic exercise or even a combination of the two^51^. To our knowledge, the effects of high-volume resistance training on muscle oxygenation have never been described. And, the benefits for chronic low back pain have never been investigated.

### Study limitations

Our study has several strengths: we used multiple specific techniques simultaneously to assess the responses to exercise, we employed a standardised exercise and we compared the population with CLBP with a well-matched control group, notably concerning the physical activity level. However, some limitations must be underlined. First, because of the variability of the participants with CLBP—due to the multifactorial causes of the pathology—our results should be considered with caution. We did not consider psychosocial and/or behavioural factors in this study. Moreover, we did not assess central sensitisation, which is commonly associated with CLBP^52^. The skin fold thickness could not be indicated in this study because it was not correctly reported during the experiments. However, it is a factor to be carefully considered when performing NIRS measurements^37^. Furthermore, we did not assess muscle activity during the protocol, whereas specific motor patterns have often been associated with CLBP^8,17,44^. Motor patterns may influence paraspinal muscle involvement to exercise and then influence the oxygenation needs in paraspinal muscle to exercise. Finally, we did not perform muscle imaging; the composition of the low back muscles may differ between the groups and partly explain the differences revealed in this study. Further investigation is needed to conclude the causes involving the metabolic differences revealed in our results. Although we found significant differences with medium and large effect sizes, the lack of information about the minimum clinically important differences in mechanical efficiency and tissue oxygenation should be considered and could be the subject of future work.

## Conclusions

We evaluated aerobic metabolic responses in the paraspinal muscles of people with CLBP. The results showed lower back weakness and reduced mechanical efficiency in participants with CLBP, even though they were physically active. We also found altered muscle oxygenation and reduced microvascular blood volume to exercise. These changes could be related to pain, and improving the aerobic metabolic response of the paraspinal muscles to exercise could be a way to reduce the muscle pain and fatigue that characterise individuals with CLBP. Further investigation is needed to explore the potential causal relationship between muscle metabolic responses and symptoms of CLBP.

## Data Availability

The datasets generated during and/or analysed during the current study are available from the corresponding author on reasonable request.

## Abbreviations

CI: Confidence interval
CLBP: Chronic low back pain
HbO_2_: Oxy-haemoglobin/myoglobin
HHb: Deoxy-haemoglobin/myoglobin
THb: Total haemoglobin/myoglobin
TOI: Tissue oxygenation index
V̇O_2_: Oxygen uptake
V̇CO_2_: Carbon dioxide output
Fr: Respiratory frequency
Vt: Tidal volume
HR: Heart rate
RER: Respiratory exchange ratio
SD: Standard deviation
SE: Standard error
V̇E: Minute ventilation
